# sFlt‐1/PlGF ratio thresholds for diagnosing pre‐eclampsia in pregnant women with high blood pressure

**DOI:** 10.1002/uog.70075

**Published:** 2025-09-24

**Authors:** X. Pan, J. Peng, Y. Chen, X. Di, P. Li, G. Zhang, H. Liu

**Affiliations:** ^1^ Department of Obstetrics First Affiliated Hospital of Jinan University Guangzhou China; ^2^ Department of Obstetrics, Guangzhou Women and Children's Medical Center Guangzhou Medical University Guangzhou China; ^3^ Maternal and Child Health Hospital of Dongguan Dongguan China

**Keywords:** adverse maternal outcome, adverse perinatal outcome, HDP, hypertension, hypertensive disorders of pregnancy, pre‐eclampsia, sFlt‐1/PlGF ratio

## Abstract

**Objective:**

An imbalance between soluble fms‐like tyrosine kinase‐1 (sFlt‐1) and placental growth factor (PlGF) is characteristic of the progression of hypertensive disorder of pregnancy (HDP) to pre‐eclampsia (PE). Monitoring the sFlt‐1/PlGF ratio to determine whether HDP progresses to PE can aid clinical management and decision‐making. This study aimed to determine the diagnostic thresholds of the sFlt‐1/PlGF ratio for early‐onset and late‐onset PE in pregnant Chinese women with high blood pressure.

**Methods:**

This single‐center, prospective, observational cohort study was conducted among pregnant women with high blood pressure (systolic blood pressure ≥ 140 mmHg and/or diastolic blood pressure ≥ 90 mmHg) at a tertiary hospital in Southern China, from January 2020 to December 2023. Women with a singleton pregnancy and complete follow‐up records were assigned to the derivation cohort or the validation cohort depending on their date of enrolment. Initial cut‐offs of the sFlt‐1/PlGF ratio to predict the development of early‐onset or late‐onset PE within 1 week after biomarker measurement were determined using receiver‐operating‐characteristics‐curve analysis in the derivation cohort. This analysis was performed separately for pregnancies with gestational age (GA) < 34 weeks and those with GA ≥ 34 weeks at the time of biomarker measurement. Subsequently, the derived cut‐offs were validated in the validation cohort. The rate of adverse maternal and perinatal outcomes was compared according to whether the sFlt‐1/PlGF ratio was above or below the validated cut‐off, stratified by GA at biomarker measurement, in both the derivation and validation cohorts.

**Results:**

A total of 1329 women with a singleton pregnancy complicated by high blood pressure, presenting between 24 + 0 and 38 + 6 weeks' gestation, were recruited during the study period. Participants were stratified into the derivation (*n* = 814 (61.2%)) and validation (*n* = 515 (38.8%)) cohorts, which had comparable PE incidence within 1 week after sFlt‐1/PlGF measurement (35.5% *vs* 38.6%, respectively; *P* = 0.267). In the derivation cohort, the optimal sFlt‐1/PlGF ratio cut‐offs were determined to be 74 for predicting early‐onset PE (diagnosis < 34 weeks) and 95 for predicting late‐onset PE (diagnosis ≥ 34 weeks). In the validation cohort, the predetermined sFlt‐1/PlGF ratio cut‐off of ≥ 74 showed a sensitivity of 87.7% (95% CI, 77.9–94.2%) and a specificity of 97.0% (95% CI, 91.6–99.4%) for predicting early‐onset PE within 1 week after biomarker measurement, while a sFlt‐1/PlGF ratio of ≥ 95 demonstrated a sensitivity of 36.5% (95% CI, 28.1–45.6%) and a specificity of 95.0% (95% CI, 91.0–97.4%) for the prediction of late‐onset PE within 1 week. Additionally, these ratios (≥ 74 for GA < 34 weeks and ≥ 95 for GA ≥ 34 weeks at biomarker measurement) significantly predicted adverse maternal and perinatal outcomes in both cohorts.

**Conclusions:**

In pregnant women with high blood pressure presenting between 24 + 0 and 38 + 6 weeks' gestation, the validated sFlt‐1/PlGF ratio cut‐offs for predicting early‐onset PE and late‐onset PE diagnosis within 1 week after biomarker measurement were 74 and 95, respectively. Furthermore, sFlt‐1/PlGF ratios ≥ 74 and ≥ 95 were associated with increased risks of adverse maternal and perinatal outcomes, suggesting clinical utility for these cut‐offs for risk stratification in Chinese women with a singleton pregnancy and high blood pressure. © 2025 The Author(s). *Ultrasound in Obstetrics & Gynecology* published by John Wiley & Sons Ltd on behalf of International Society of Ultrasound in Obstetrics and Gynecology.

## INTRODUCTION

Hypertensive disorders of pregnancy (HDP) refer to a group of conditions characterized by elevated blood pressure during pregnancy, and are a significant cause of maternal and neonatal adverse outcome^1^, ^2^. Pre‐eclampsia (PE) is a progressive, multisystem disorder characterized primarily by organ dysfunction, making its early identification crucial for the clinical management of HDP. Imbalances between soluble fms‐like tyrosine kinase‐1 (sFlt‐1) and placental growth factor (PlGF) are hallmark features of PE progression and typically occur before the clinical symptoms of PE become apparent^3^; therefore, the sFlt‐1/PlGF ratio has been proposed as a biomarker for predicting PE^4^. Previous studies, including the PROGNOSIS and PROGNOSIS Asia trials, validated the negative predictive value (NPV) of sFlt‐1/PlGF ≤ 38 for ruling out PE within 1 week after sFlt‐1/PlGF biomarker measurement among women with suspected PE^5^, ^6^. Verlohren *et al*.^7^ proposed that a sFlt‐1/PlGF ratio ≥ 85 and sFlt‐1/PlGF ratio ≥ 110 can be used as diagnostic tools to assist in the diagnosis of early‐onset PE and late‐onset PE, respectively, but there are conflicting results regarding the optimal cut‐off value of the sFlt‐1/PlGF ratio for diagnosing PE^1^, ^7^, ^8^. Currently, limited research exists on optimal cut‐off values for diagnosing PE, especially in a Chinese population with high blood pressure. This study aimed to determine the diagnostic thresholds of the sFlt‐1/PlGF ratio for early‐onset and late‐onset PE in a large cohort of pregnant women with high blood pressure in Southern China, and to assess their association with adverse maternal and perinatal outcomes.

## METHODS

### Study population

This single‐center, prospective, observational cohort study included women with a singleton pregnancy and high maternal blood pressure who underwent prenatal care and delivery at the Zhujiang New Town Campus of Guangzhou Women and Children's Medical Center, Guangzhou Medical University, Guangzhou, China, between January 2020 and December 2023, with complete follow‐up records. As part of one of the largest tertiary maternal–child hospitals in Southern China (approximately 30 000 annual deliveries), Zhujiang New Town Campus handles 14 000–16 000 deliveries annually, with a documented prevalence of HDP of 5.4–6.1% per year. Participants provided written informed consent, and the study was approved by the Ethics Committee of Guangzhou Women and Children's Medical Center (approval No.: 2017022008).

Inclusion criteria included: (1) maternal age ≥ 18 years; (2) HDP diagnosed according to International Society for the Study of Hypertension in Pregnancy (ISSHP) guidelines^9^, including chronic hypertension, white‐coat hypertension, masked hypertension, transient gestational hypertension and gestational hypertension, all uniformly defined by clinic‐measured systolic blood pressure ≥ 140 mmHg and/or diastolic blood pressure ≥ 90 mmHg; (3) singleton pregnancy between 24 + 0 and 38 + 6 weeks' gestation; and (4) delivery occurred at the study hospital with complete follow‐up data available.

Exclusion criteria included: (1) pregnancy loss due to fetal anomaly or chromosomal abnormality; (2) confirmed PE or HELLP syndrome diagnosis before enrolment; (3) foreign nationality (due to differences in follow‐up, healthcare access or baseline characteristics); and (4) incomplete records or loss to follow‐up.

### Study design

We conducted a two‐phase cohort study to determine and validate sFlt‐1/PlGF ratio thresholds for predicting early‐onset PE and late‐onset PE within 1 week after the measurement of sFlt‐1 and PlGF. Participants were assigned to one cohort based on the date of enrolment within the study period. Within all cohorts, further stratification by gestational age (GA) at biomarker measurement (< 34 weeks or ≥ 34 weeks) was performed to establish GA‐specific cut‐off values.

Participants enrolled between January 2020 and July 2022 were assigned to the derivation cohort for the initial determination of optimal sFlt‐1/PlGF ratio cut‐offs for predicting PE diagnosis within 1 week after biomarker measurement. The validation cohort included participants enrolled between August 2022 and December 2023, in which the derived cut‐offs were applied to assess their ability to predict PE diagnosis within 1 week of biomarker measurement.

The sample size was calculated using PASS 2021 version 21.0.3 (NCSS LLC, Kaysville, UT, USA) based on an expected sensitivity of 76%^7^ and an assumed PE prevalence of 20% among women with high blood pressure^10^. A minimum of 620 participants (310 per cohort) was required to achieve a target significance level of 0.05 (two‐tailed) and a power of 90% (β = 0.10).

### Data collection

Demographic and clinical data were collected, including maternal age, gravidity, parity, body mass index (BMI), blood pressure, laboratory assessment of organ function, neonatal outcomes and time interval between sFlt‐1/PlGF sampling and PE diagnosis. Maternal serum concentrations of sFlt‐1 and PlGF were measured using an automated immunoassay platform (Cobas e411; Roche Diagnostics, Indianapolis, IN, USA) in the hospital's central laboratory. The sFlt‐1/PlGF ratio was calculated for each sample.

### Diagnostic criteria

The diagnosis of HDP was based on ISSHP guideline recommendations^9^. PE was defined as new‐onset hypertension (≥ 140/90 mmHg) after 20 weeks' gestation, accompanied by one or more of the following new conditions: proteinuria, defined as ≥ 0.3 g per 24‐h urine collection, urine protein‐to‐creatinine ratio > 0.3 or random dipstick ≥ 2+; neurological complications (eclampsia, severe headache, visual disturbances, stroke); pulmonary edema; hematological abnormalities (platelet count < 150 × 10^9^/L, disseminated intravascular coagulation (DIC), hemolysis); acute kidney injury, defined as creatinine ≥ 90 μmol/L or 1 mg/dL; liver dysfunction, indicated by alanine aminotransferase (ALT) or aspartate aminotransferase (AST) > 40 IU/L, with or without right upper quadrant pain; or uteroplacental dysfunction (placental abruption, fetal growth restriction, abnormal umbilical artery Doppler indices or intrauterine fetal death). Besides PE, other hypertensive conditions during pregnancy were categorized as follows: (1) hypertension diagnosed before pregnancy or newly discovered before 20 weeks' gestation, including chronic hypertension, white‐coat hypertension and masked hypertension; and (2) hypertension occurring after 20 weeks, including transient hypertension of pregnancy and pregnancy‐induced hypertension. Early‐onset PE was defined as PE diagnosed < 34 weeks and late‐onset PE was defined as PE diagnosed ≥ 34 weeks^11^.

PE diagnosed within 1 week refers to PE clinically confirmed within 7 days after biomarker measurement. In both cohorts (derivation and validation), pregnancies were stratified by GA at biomarker measurement (< 34 or ≥ 34 weeks). Cases were categorized as PE (clinically diagnosed within 1 week, with GA‐based subclassification) or non‐PE (no PE diagnosis within 1 week).

Adverse maternal outcome was defined as the occurrence of at least one of the following^12^, ^13^: liver dysfunction (ALT/AST > 80 IU/L); platelet count < 100 × 10^9^/L; DIC; placental abruption; pulmonary edema; neurological complications; renal impairment (creatinine > 97 μmol/L or 1.1 mg/dL); or maternal death. Adverse fetal and neonatal outcomes included: small‐for‐gestational age (SGA) with birth weight < 10^th^ percentile^14^; neonatal asphyxia based on 5‐min Apgar score < 7^15^, ^16^; neonatal intensive care unit (NICU) admission for 48 h; or perinatal death, including fetal death^17^ and early neonatal death^18^ (death of a liveborn baby within 7 days after birth).

### Statistical analysis

Statistical analysis was performed using SPSS version 22.0 (Armonk, NY, USA). Continuous data are presented as mean ± SD or median (interquartile range) depending on normality, which was assessed using the Shapiro–Wilk test. Group comparisons were conducted using independent sample *t*‐tests or the Mann–Whitney *U*‐test, as appropriate. Categorical data are presented as *n* (%), and comparisons were made using the chi‐square test or Fisher's exact test.

Receiver‐operating‐characteristics (ROC)‐curve analysis was used to evaluate the diagnostic performance of the sFlt‐1/PlGF ratio for early‐onset and late‐onset PE. The area under the ROC curve (AUC) with 95% CI was used to quantify the discriminative ability. Calibration was assessed using the Hosmer–Lemeshow test with 10 quantile‐based groups. Optimal diagnostic thresholds for the sFlt‐1/PlGF ratio were determined in the derivation cohort using ROC‐curve analysis combined with sensitivity analysis at fixed specificity levels. To align with clinical priorities, we preset a target specificity of approximately 95% to minimize false positives. Diagnostic accuracy metrics, including sensitivity, specificity, positive predictive value (PPV), NPV, positive likelihood ratio (LR+) and negative likelihood ratio (LR–), were calculated with 95% CIs. The established thresholds were then independently validated in the validation cohort. Adverse maternal and perinatal outcomes were compared according to whether the sFlt‐1/PlGF ratio was above or below the validated cut‐off in both cohorts, stratified by GA at biomarker measurement, using the chi‐square or Fisher's exact test, with crude odds ratios (ORs) and 95% CIs calculated for risk associations. No adjustment was made for multiple testing. *P* < 0.05 (two‐tailed) was considered statistically significant.

## RESULTS

### Baseline characteristics

**Figure 1:**
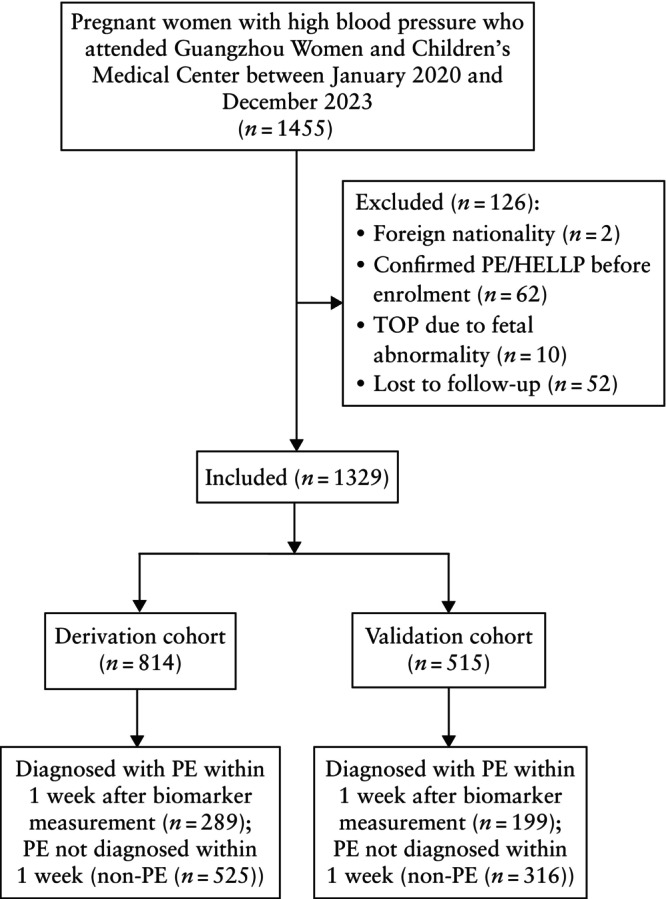
Flowchart summarizing inclusion of women with singleton pregnancy complicated by high blood pressure in derivation cohort or in validation cohort for determination of optimal soluble fms‐like tyrosine kinase‐1/placental growth factor ratio cut‐offs for predicting pre‐eclampsia (PE) within 1 week after biomarker measurement. TOP, termination of pregnancy.

From January 2020 to December 2023, a total of 1329 eligible pregnant women with high blood pressure, between 24 + 0 and 38 + 6 weeks' gestation, were recruited, of whom 814 (61.2%) were assigned to the derivation cohort and 515 (38.8%) were assigned to the validation cohort (Figure 1). The incidence of PE diagnosis within 1 week after sFlt‐1/PlGF measurement was comparable between the two cohorts (35.5% in the derivation cohort *vs* 38.6% in the validation cohort; *P* = 0.267). No significant differences were observed between the derivation and validation cohorts for maternal age, median GA at recruitment, prepregnancy BMI, rate of *in‐vitro* fertilization or the proportion of women with pre‐existing chronic hypertension, pre‐existing diabetes mellitus or gestational diabetes mellitus (Table 1).

**Table 1:** Baseline demographic characteristics of women with singleton pregnancy and high blood pressure, according to inclusion in derivation cohort or in validation cohort for determination of optimal soluble fms‐like tyrosine kinase‐1/placental growth factor ratio cut‐offs for predicting pre‐eclampsia (PE) within 1 week after biomarker measurement

Characteristic	Derivation cohort (*n* = 814)	Validation cohort (*n* = 515)	*P*
Maternal age (years)	31.8 ± 4.6	32.2 ± 4.7	0.131
Gravidity	2 (1–3)	2 (1–3)	0.563
Parity	0 (0–1)	0 (0–1)	0.09
Prepregnancy BMI (kg/m^2^)	22.7 ± 3.9	23.1 ± 3.9	0.126
GA at recruitment (weeks)	36.0 (33.4–37.6)	35.6 (32.4–37.6)	0.08
IVF	71 (8.7)	54 (10.5)	0.283
Pre‐existing hypertension	118 (14.5)	82 (15.9)	0.479
Pre‐existing DM	21 (2.6)	9 (1.7)	0.320
GDM	211 (25.9)	147 (28.5)	0.294
PE incidence	289 (35.5)	199 (38.6)	0.267

Data are shown as mean ± SD, median (interquartile range) or *n* (%). BMI, body mass index; DM, diabetes mellitus; GA, gestational age; GDM, gestational diabetes mellitus; IVF, *in‐vitro* fertilization.

### sFlt‐1/PlGF ratio in PE *vs* non‐PE groups

**Figure 2:**
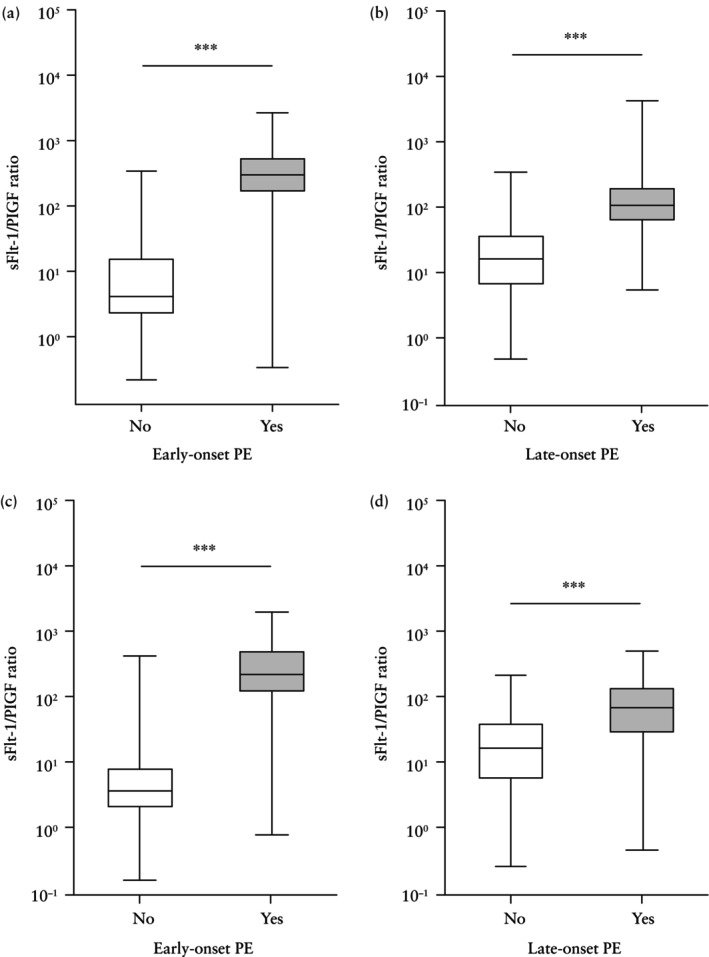
Comparison of soluble fms‐like tyrosine kinase‐1 (sFlt‐1)/placental growth factor (PlGF) ratio in pregnant women with high blood pressure according to whether they developed pre‐eclampsia (PE) within 1 week after biomarker measurement at < 34 weeks' gestation (a,c) or at ≥ 34 weeks' gestation (b,d), in derivation cohort (*n* = 814) (a,b) and in validation cohort (*n* = 515) (c,d). Median, interquartile range and range are shown. *y*‐axis is log_10_‐scaled. ***P < 0.001. Early‐onset PE was defined as PE diagnosed < 34 weeks and late‐onset PE as PE diagnosed ≥ 34 weeks.

In the derivation cohort, among 250 pregnancies with GA < 34 weeks at sFlt‐1 and PlGF measurement, 111 (44.4%) developed early‐onset PE within 1 week after biomarker measurement, with a significantly higher median sFlt‐1/PlGF ratio in early‐onset PE cases *vs* non‐PE cases (304.8 *vs* 4.2; *P* < 0.001) (Figure 2a). Similarly, among 564 pregnancies with GA ≥ 34 weeks at biomarker measurement, 31.6% (178/564) developed late‐onset PE within 1 week, with a significantly higher median sFlt‐1/PlGF ratio in late‐onset PE cases *vs* non‐PE cases (109.2 *vs* 16.8; *P* < 0.001) (Figure 2b).

The validation cohort comprised 174 pregnancies with GA < 34 weeks and 341 pregnancies with GA ≥ 34 weeks at sFlt‐1 and PlGF measurement, of which 42.0% (73/174) and 37.0% (126/341) were diagnosed with PE within 1 week after measurement, respectively. Consistent with the derivation cohort, the median sFlt‐1/PlGF ratio was significantly higher in PE cases *vs* non‐PE controls for early‐onset PE (215.6 *vs* 3.7; *P* < 0.001) and late‐onset PE (67.2 *vs* 16.8; *P* < 0.001) (Figure 2c,d).

### Diagnostic performance of sFlt‐1/PlGF ratio for PE within 1 week

**Figure 3:**
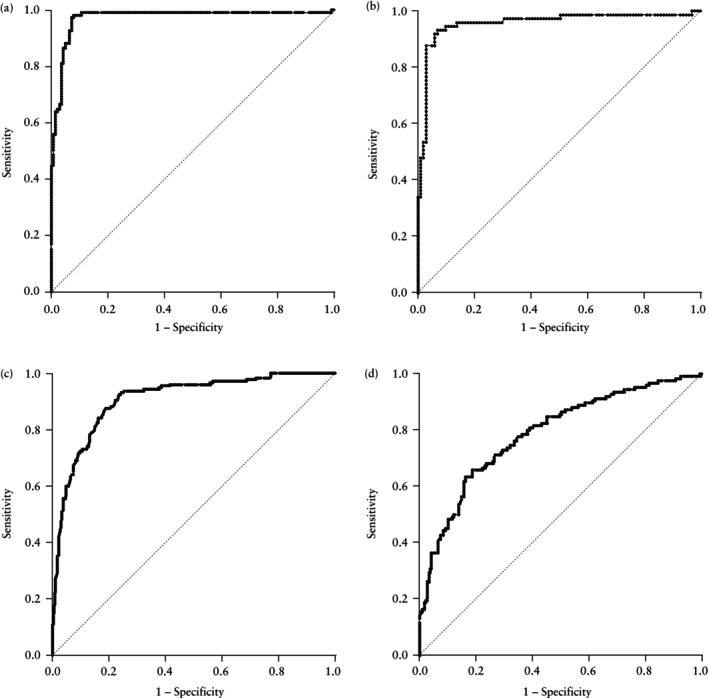
Receiver‐operating‐characteristics curves showing ability of soluble fms‐like tyrosine kinase‐1/placental growth factor ratio measured at < 34 weeks' gestation (a,b) or ≥ 34 weeks' gestation (c,d) to predict pre‐eclampsia within 1 week after biomarker measurement in pregnant women with high blood pressure, in the derivation (a,c) and validation (b,d) cohorts.

In the derivation cohort, in women with GA < 34 weeks at biomarker measurement, the sFlt‐1/PlGF ratio demonstrated excellent diagnostic accuracy for early‐onset PE within 1 week after assessment, with an AUC of 0.97 (95% CI, 0.94–0.99) (Figure 3a). Calibration was good, as shown by the Hosmer–Lemeshow test (*P* = 0.202). ROC‐curve analysis combined with fixed‐specificity analysis (Table S1) identified an optimal sFlt‐1/PlGF ratio cut‐off value of 74, yielding a sensitivity of 95.5% (95% CI, 89.8–98.5%), specificity of 92.8% (95% CI, 87.2–96.5%) and LR+ of 13.27 (95% CI, 7.30–24.15) (Table 2).

**Table 2:** Diagnostic performance of soluble fms‐like tyrosine kinase‐1 (sFlt‐1)/placental growth factor (PlGF) ratio thresholds to predict pre‐eclampsia within 1 week after biomarker measurement in pregnancies with gestational age < 34 weeks at assessment

Cohort	sFlt‐1/PlGF cut‐off	Sensitivity (%)	Specificity (%)	LR+	LR−	PPV (%)	NPV (%)
Derivation	74	95.5 (89.8–98.5)	92.8 (87.2–96.5)	13.27 (7.30–24.15)	0.05 (0.02–0.11)	91.4 (85.4–95.1)	96.3 (91.6–98.4)
	85	92.8 (86.3–96.8)	92.8 (87.2–96.5)	12.90 (7.08–23.49)	0.08 (0.04–0.15)	91.2 (85.0–94.9)	94.2 (89.2–96.9)
Validation	74	87.7 (77.9–94.2)	97.0 (91.6–99.4)	29.52 (9.65–90.28)	0.13 (0.07–0.23)	95.5 (87.5–98.5)	91.6 (85.5–95.3)
	85	84.9 (74.6–92.2)	97.0 (91.6–99.4)	28.59 (9.34–87.53)	0.16 (0.09–0.27)	95.4 (87.1–98.4)	89.9 (83.8–93.9)

Values in parentheses are 95% CI. LR+, positive likelihood ratio; LR−, negative likelihood ratio; NPV, negative predictive value; PPV, positive predictive value.

The validation cohort with biomarker measurement at < 34 weeks' gestation further confirmed the diagnostic accuracy of using a sFlt‐1/PlGF ratio of ≥ 74 for predicting early‐onset PE within 1 week after measurement, with an AUC of 0.96 (95% CI, 0.91–0.98) (Figure 3b). The Hosmer–Lemeshow test indicated good calibration (*P* = 0.119). Diagnostic performance metrics included a sensitivity of 87.7% (95% CI, 77.9–94.2%), specificity of 97.0% (95% CI, 91.6–99.4%) and LR+ of 29.52 (95% CI, 9.65–90.28) (Table 2). Notably, applying a higher cut‐off of 85^7^ yielded a specificity of 97.0% (95% CI, 91.6–99.4%) with a sensitivity of 84.9% (95% CI, 74.6–92.2%) in this cohort. Restricting the analysis to women with pre‐existing hypertension (*n* = 40), a sFlt‐1/PlGF ratio of ≥ 74 showed sensitivity and specificity of 84.2% (95% CI, 68.7–94.0%) and 96.3% (95% CI, 87.3–99.5%), respectively.

Similarly, in women with GA ≥ 34 weeks at biomarker measurement in the derivation cohort, the sFlt‐1/PlGF ratio demonstrated high diagnostic accuracy for late‐onset PE within 1 week after measurement, with an AUC of 0.91 (95% CI, 0.88–0.93) (Figure 3c). The Hosmer–Lemeshow test indicated good calibration (*P* = 1.000). ROC‐curve analysis combined with fixed‐specificity analysis (Table S2) identified an optimal sFlt‐1/PlGF ratio cut‐off value of 95, yielding a sensitivity of 60.1% (95% CI, 52.5–67.4%), specificity of 95.0% (95% CI, 92.4–97.0%) and LR+ of 12.21 (95% CI, 7.75–19.24) (Table 3).

**Table 3:** Diagnostic performance of soluble fms‐like tyrosine kinase‐1 (sFlt‐1)/placental growth factor (PlGF) ratio thresholds to predict pre‐eclampsia within 1 week after biomarker measurement in pregnancies with gestational age ≥ 34 weeks at assessment

Cohort	sFlt‐1/PlGF cut‐off	Sensitivity (%)	Specificity (%)	LR+	LR−	PPV (%)	NPV (%)
Derivation	95	60.1 (52.5–67.4)	95.0 (92.4–97.0)	12.21 (7.75–19.24)	0.42 (0.35–0.50)	85.0 (78.1–89.9)	83.8 (81.2–86.1)
	110	48.9 (41.3–56.5)	96.6 (94.3–98.2)	14.51 (8.33–25.28)	0.53 (0.46–0.61)	87.0 (79.3–92.1)	80.4 (78.0–82.6)
Validation	95	36.5 (28.1–45.6)	95.0 (91.0–97.4)	7.14 (3.84–13.26)	0.67 (0.58–0.77)	80.7 (69.2–88.6)	71.8 (69.0–74.5)
	110	33.3 (25.2–42.3)	95.8 (92.2–98.1)	7.96 (4.01–15.81)	0.70 (0.61–0.79)	82.4 (70.2–90.3)	71.0 (68.4–73.6)

Values in parentheses are 95% CI. LR+, positive likelihood ratio; LR−, negative likelihood ratio; NPV, negative predictive value; PPV, positive predictive value.

In the validation cohort, in women with GA ≥ 34 weeks at biomarker measurement, a sFlt‐1/PlGF ratio ≥ 95 yielded an AUC of 0.79 (95% CI, 0.74–0.83) (Figure 3d), with a sensitivity of 36.5% (95% CI, 28.1–45.6%) and a specificity of 95.0% (95% CI, 91.0–97.4%) (Table 3). The Hosmer–Lemeshow test indicated good calibration (*P* = 0.070). Using a higher cut‐off of 110^7^, the specificity improved slightly to 95.8% (95% CI, 92.2–98.1%), but sensitivity declined further to 33.3% (95% CI, 25.2–42.3%). Subgroup analysis of patients with chronic hypertension (*n* = 42) showed a sensitivity of 29.7% (95% CI, 15.9–47.0%) and a specificity of 95.6% (95% CI, 88.1–99.1%) using a sFlt‐1/PlGF ratio cut‐off of 95.

### Adverse outcome based on sFlt‐1/PlGF ratio

Women who underwent biomarker measurement at < 34 weeks from both derivation and validation cohorts were stratified using the predefined sFlt‐1/PlGF ratio cut‐off of 74 for predicting PE diagnosis to determine the rate of adverse outcome. Compared with a sFlt‐1/PlGF ratio of < 74, a sFlt‐1/PlGF ratio ≥ 74 was associated with a significantly increased risk of adverse maternal outcome (derivation: 3.8% *vs* 29.9%, OR, 10.93 (95% CI, 4.11–29.03), *P* < 0.001; validation: 4.7% *vs* 17.9%, OR, 4.45 (95% CI, 1.49–13.29), *P* = 0.004), including placental abruption (derivation: 1.5% *vs* 8.5%, OR, 6.12 (95% CI, 1.31–28.54), *P* = 0.015; validation: 0.9% *vs* 10.4%, OR, 12.37 (95% CI, 1.49–102.93), *P* = 0.006), and a significantly higher rate of perinatal adverse outcome (derivation: 20.3% *vs* 90.6%, OR, 37.83 (95% CI, 17.85–80.17), *P* < 0.001; validation: 15.9% *vs* 88.1%, OR, 39.04 (95% CI, 15.84–96.24); *P* < 0.001). There was a consistent perinatal mortality rate of 6.0% in both the derivation and validation cohorts for sFlt‐1/PlGF ratio ≥ 74 (7/117 and 4/67, respectively) (Table 4).

**Table 4:** Comparison of adverse pregnancy outcomes in derivation and validation cohorts between women with soluble fms‐like tyrosine kinase‐1 (sFlt‐1)/placental growth factor (PlGF) ratio < 74 and those with sFlt‐1/PlGF ratio ≥ 74 at < 34 weeks' gestation

	Derivation cohort				Validation cohort			
Variable	sFlt‐1/PlGF < 74 (*n* = 133)	sFlt‐1/PlGF ≥ 74 (*n* = 117)	*P*	OR (95% CI)	sFlt‐1/PlGF < 74 (*n* = 107)	sFlt‐1/PlGF ≥ 74 (*n* = 67)	*P*	OR (95% CI)
Adverse maternal outcome*	5 (3.8)	35 (29.9)	< 0.001	10.93 (4.11–29.03)	5 (4.7)	12 (17.9)	0.004	4.45 (1.49–13.29)
Eclampsia	0 (0)	1 (0.9)	0.468	—	1 (0.9)	3 (4.5)	0.160	4.97 (0.51–48.79)
Placental abruption	2 (1.5)	10 (8.5)	0.015	6.12 (1.31–28.54)	1 (0.9)	7 (10.4)	0.006	12.37 (1.49–102.93)
Pulmonary edema	0 (0)	5 (4.3)	0.021	—	2 (1.9)	2 (3.0)	0.640	1.62 (0.22–11.75)
PLT < 100 × 10^9^/L	1 (0.8)	12 (10.3)	< 0.001	15.09 (1.93–117.89)	2 (1.9)	3 (4.5)	0.374	2.46 (0.40–15.13)
Renal impairment	1 (0.8)	3 (2.6)	0.343	3.47 (0.36–33.86)	0 (0)	2 (3.0)	0.147	—
Liver dysfunction	2 (1.5)	14 (12.0)	< 0.001	8.90 (1.98–40.06)	1 (0.9)	2 (3.0)	0.560	3.26 (0.29–36.69)
Perinatal adverse outcome†	27 (20.3)	106 (90.6)	< 0.001	37.83 (17.85–80.17)	17 (15.9)	59 (88.1)	< 0.001	39.04 (15.84–96.24)
SGA	20 (15.0)	64 (54.7)	< 0.001	6.82 (3.75–12.42)	17 (15.9)	59 (88.1)	< 0.001	39.04 (15.84–96.24)
Neonatal asphyxia	5 (3.8)	26 (22.2)	< 0.001	7.31 (2.71–19.77)	3 (2.8)	10 (14.9)	0.006	6.08 (1.61–23.00)
NICU admission	21 (15.8)	102 (87.2)	< 0.001	36.27 (17.74–74.12)	20 (18.7)	62 (92.5)	< 0.001	53.94 (19.21–151.50)
Perinatal death	2 (1.5)	7 (6.0)	0.087	4.17 (0.85–20.48)	2 (1.9)	4 (6.0)	0.206	3.33 (0.59–18.73)

Data are shown as *n* (%), unless stated otherwise. *P* indicates comparison between *n* (%). Odds ratio (OR) was not estimated when the outcome was not observed in one group.

* Adverse maternal outcome was defined as the occurrence of at least one of the following: liver dysfunction; platelet (PLT) count < 100 × 10^9^/L; disseminated intravascular coagulation; placental abruption; pulmonary edema; neurological complications; renal impairment; maternal death.

† Perinatal adverse outcome included: small‐for‐gestational age (SGA) with birth weight < 10^th^ percentile; neonatal asphyxia based on 5‐min Apgar score < 7; neonatal intensive care unit (NICU) admission for at least 48 h; and perinatal death, including fetal death and early neonatal death.

Women who underwent biomarker measurement at ≥ 34 weeks from both the derivation and validation cohorts were stratified using the predefined sFlt‐1/PlGF cut‐off of 95 for predicting PE diagnosis to determine the rate of adverse outcome. Compared with a sFlt‐1/PlGF ratio of < 95, a sFlt‐1/PlGF ratio ≥ 95 significantly increased the risk of adverse maternal outcome (derivation: 9.4% *vs* 18.8%, OR, 2.22 (96% CI, 1.29–3.85), *P* = 0.004; validation: 7.1% *vs* 23.0%, OR, 3.87 (95% CI, 1.83–8.20), *P* < 0.001), including renal impairment (derivation: 0.5% *vs* 3.1%, OR, 7.00 (95% CI, 1.27–38.67), *P* = 0.026; validation: 0.4% *vs* 4.9%, OR, 14.43 (95% CI, 1.48–141.20), *P* = 0.019), and significantly increased the rate of adverse perinatal outcome (derivation: 13.3% *vs* 35.9%, OR, 3.66 (95% CI, 2.32–5.76), *P* < 0.001; validation: 12.9% *vs* 39.3%, OR, 4.40 (95% CI, 2.36–8.19); *P* < 0.001). The perinatal mortality rates for sFlt‐1/PlGF ratio ≥ 95 in the derivation and validation cohorts were 1.6% (2/128) and 3.3% (2/61), respectively (Table 5).

**Table 5:** Comparison of adverse pregnancy outcomes in derivation and validation cohorts between women with soluble fms‐like tyrosine kinase‐1 (sFlt‐1)/placental growth factor (PlGF) ratio < 95 and those with sFlt‐1/PlGF ratio ≥ 95 at ≥ 34 weeks' gestation

	Derivation cohort				Validation cohort			
Variable	sFlt‐1/PlGF < 95 (*n* = 436)	sFlt‐1/PlGF ≥ 95 (*n* = 128)	*P*	OR (95% CI)	sFlt‐1/PlGF < 95 (*n* = 280)	sFlt‐1/PlGF ≥ 95 (*n* = 61)	*P*	OR (95% CI)
Adverse maternal outcome*	41 (9.4)	24 (18.8)	0.004	2.22 (1.29–3.85)	20 (7.1)	14 (23.0)	< 0.001	3.87 (1.83–8.20)
Eclampsia	1 (0.2)	1 (0.8)	0.403	3.43 (0.21–55.15)	0 (0)	0 (0)	—	—
Placental abruption	10 (2.3)	6 (4.7)	0.220	2.10 (0.75–5.88)	10 (3.6)	3 (4.9)	0.710	1.40 (0.37–5.23)
PLT < 100 × 10^9^/L	16 (3.7)	9 (7.0)	0.104	1.99 (0.86–4.61)	7 (2.5)	6 (9.8)	0.007	4.26 (1.38–13.15)
Pulmonary edema	6 (1.4)	4 (3.1)	0.246	2.31 (0.64–8.32)	1 (0.4)	2 (3.3)	0.084	9.46 (0.84–106.02)
Renal impairment	2 (0.5)	4 (3.1)	0.026	7.00 (1.27–38.67)	1 (0.4)	3 (4.9)	0.019	14.43 (1.48–141.20)
Liver dysfunction	9 (2.1)	6 (4.7)	0.105	2.33 (0.82–6.68)	3 (1.1)	1 (1.6)	0.547	1.54 (0.16–15.05)
Perinatal adverse outcome†	58 (13.3)	46 (35.9)	< 0.001	3.66 (2.32–5.76)	36 (12.9)	24 (39.3)	< 0.001	4.40 (2.36–8.19)
SGA	56 (12.8)	45 (35.2)	< 0.001	3.68 (2.33–5.82)	35 (12.5)	24 (39.3)	< 0.001	4.54 (2.43–8.47)
Neonatal asphyxia	1 (0.2)	4 (3.1)	0.011	14.03 (1.55–126.69)	2 (0.7)	1 (1.6)	0.447	2.32 (0.21–25.97)
NICU admission	8 (1.8)	31 (24.2)	< 0.001	17.10 (7.62–38.35)	17 (6.1)	13 (21.3)	< 0.001	4.19 (1.91–9.19)
Perinatal death	2 (0.5)	2 (1.6)	0.223	3.44 (0.48–24.70)	1 (0.4)	2 (3.3)	0.084	9.46 (0.84–106.02)

Data are given as *n* (%), unless stated otherwise. *P* indicates comparison between *n* (%). Odds ratio (OR) was not estimated when the outcome was not observed in one group.

* Adverse maternal outcome was defined as the occurrence of at least one of the following: liver dysfunction; platelet (PLT) count < 100 × 10^9^/L; disseminated intravascular coagulation; placental abruption; pulmonary edema; neurological complications; renal impairment; maternal death.

† Perinatal adverse outcome included: small‐for‐gestational age (SGA) with birth weight < 10^th^ percentile; neonatal asphyxia based on 5‐min Apgar score < 7; neonatal intensive care unit (NICU) admission for at least 48 h; and perinatal death, including fetal death and early neonatal death.

## DISCUSSION

This prospective cohort study from Southern China established validated GA‐specific sFlt‐1/PlGF ratio thresholds for the prediction of PE diagnosis within 1 week of sFlt‐1 and PlGF measurement. The sFlt‐1/PlGF ratio cut‐off of ≥ 74 demonstrated balanced accuracy (sensitivity, 87.7%; specificity, 97.0%) for early‐onset PE prediction, while the sFlt‐1/PlGF ratio of ≥ 95 maintained good specificity (95.0%) but had limited sensitivity (36.5%) for late‐onset PE prediction. Our findings also demonstrated that a sFlt‐1/PlGF ratio of ≥ 74 before 34 weeks' gestation or ≥ 95 at ≥ 34 weeks is significantly associated with adverse maternal and perinatal outcomes in singleton pregnancies with high maternal blood pressure, providing clinically actionable risk stratification.

### Diagnostic value of sFlt‐1/PlGF ratio for early‐onset PE

Accurate PE differentiation within HDP enables risk‐adapted care, limiting preventable maternal–fetal mortality^19^. Approximately 10% of PE cases are diagnosed before 34 weeks, at which time the risk of severe complications and mortality are significantly higher owing to preterm delivery. The pivotal role of angiogenic imbalance in the pathogenesis of early‐onset PE is well established^1^, ^5^, ^10^, ^20^, ^21^, ^22^. Our findings corroborate this mechanism, demonstrating a significantly elevated sFlt‐1/PlGF ratio in women diagnosed with early‐onset PE.

The PROGNOSIS study established a sFlt‐1/PlGF ratio of ≤ 38 to effectively rule out PE within 1 week after biomarker measurement (NPV, > 99%), but it demonstrated limited rule‐in utility (PPV, 36.7% within 4 weeks)^5^. While valuable for short‐term exclusion, this threshold lacks optimization and clinical applicability for diagnosing early‐onset PE in hypertensive cohorts. Previous studies report varying sFlt‐1/PlGF ratio cut‐offs for the prediction of early‐onset PE^1^, ^7^, ^8^. An Iranian study reported a sFlt‐1/PlGF cut‐off of 24.96 in 38 cases of PE (sensitivity, 84.2%; specificity, 85.0%), without early/late‐onset stratification^1^. Another study reported a cut‐off of 45 for a Japanese cohort of early‐onset PE cases^8^ (< 32 weeks: sensitivity, 100%; specificity, 95%) with a limited sample size (*n* = 15), while a study in a European cohort used a sFlt‐1/PlGF ratio of ≥ 85 for early‐onset PE (< 34 weeks: sensitivity, 88%; specificity, 99.5%), which lacked predictive values^7^. Notably, these case–control studies were limited by small samples, which highlights the necessity of prospective validation.

Our prospective cohort of pregnancies with high blood pressure established a sFlt‐1/PlGF ratio threshold of 74 for predicting early‐onset PE, achieving balanced accuracy. Compared with the conventional cut‐off of 85^7^, our threshold maintained high specificity (97.0%) while improving sensitivity (87.7% *vs* 84.9%), providing clinicians with a reliable decision‐making tool for the management of early‐onset PE.

The marginally lower sensitivity in the validation cohort compared with that in the derivation cohort (87.7% *vs* 95.5%) is consistent with the PROGNOSIS study^5^, reflecting expected real‐world variability due to population heterogeneity, variations in the spectrum of disease severity and temporal patterns of biomarker elevation (PE heterogeneity) across cohorts. Clinically, our optimized population‐specific cut‐off ensures clinical reliability in pregnancies with high blood pressure, for which high specificity is critical for intervention decisions. Future multicenter studies with standardized protocols and machine‐learning‐based dynamic thresholds may further refine performance.

### Diagnostic value of sFlt‐1/PlGF ratio for late‐onset PE

Approximately 90% of PE cases are diagnosed after 34 weeks. In general, late‐onset PE cases have better maternal and fetal outcomes than do cases of early‐onset PE. Research on the best method for aiding the diagnosis of late‐onset PE is limited and existing studies report heterogeneous performance characteristics. Ohkuchi *et al*.^8^ identified a sFlt‐1/PlGF ratio cut‐off of ≥ 45 (sensitivity, 79%) in Japanese women (*n* = 19), while Verlohren *et al*.^7^ proposed a cut‐off of ≥ 110 in a European cohort, although predictive values were not reported. In our Southern Chinese cohort of women at ≥ 34 weeks' gestation at the time of measurement, a sFlt‐1/PlGF ratio of ≥ 95 demonstrated high specificity but limited sensitivity for predicting late‐onset PE within 1 week after biomarker measurement. The performance of this cut‐off remained inferior to that of the cut‐off used in early‐onset PE prediction^7^, ^8^, consistent with the attenuated angiogenic imbalance of late‐onset PE^23^, ^24^. Validation of the ≥ 110 cut‐off proposed by Verlohren *et al*.^7^ in our cohort, revealed comparable specificity (95.5% *vs* 95.0% in our cohort) but reduced sensitivity (58.2% *vs* 33.3% in our cohort), highlighting population‐specific variations. While the high specificity ensures reliable rule‐in of late‐onset PE, the low sensitivity mandates the need for combined clinical evaluation for the optimal prediction of late‐onset PE. These findings support the value of GA‐specific thresholds while underscoring the necessity of multimodal management for late‐onset PE.

### Association of sFlt‐1/PlGF ratio with adverse outcomes

HDP are a leading cause of maternal–fetal morbidity and mortality, particularly in early‐onset PE, which tends to have a higher risk of adverse outcome^16^. While clinical criteria (hypertension/proteinuria) have limited predictive value (PPV, 18%–20%) for PE‐related complications^25^, early risk stratification enables targeted interventions for high‐risk pregnancies.

The sFlt‐1/PlGF ratio demonstrates a stronger correlation with adverse maternofetal outcomes than do other biochemical markers^26^. A previous study in an Asian population with suspected PE reported a NPV of 98.9% for excluding adverse fetal outcomes within 1 week after measurement when the sFlt‐1/PlGF ratio was ≤ 38, showing short‐term clinical value^6^. In contrast, a prospective study of 176 cases presenting with suspected PE at < 34 weeks reported an association between sFlt‐1/PlGF ratio ≥ 85 and the risk of adverse outcome^27^. Notably, our optimized sFlt‐1/PlGF ratio thresholds (≥ 74 at GA < 34 weeks and ≥ 95 at GA ≥ 34 weeks) are associated with an increased risk of maternal–fetal adverse outcomes, enabling targeted monitoring.

The PRAECIS study^10^ identified significantly increased adverse maternal and perinatal outcomes using a sFlt‐1/PlGF ratio of ≥ 40 in those with HDP (at 23 + 0 to 34 + 6 weeks), although the inpatient focus and lack of GA stratification limit the generalizability of these findings. Research has demonstrated that elevated sFlt‐1/PlGF ratio in pregnant women is associated with a higher risk of uteroplacental ischemia–reperfusion injury and placental abruption^28^. Data from our study also show that, in pregnant women with high blood pressure, a sFlt‐1/PlGF ratio ≥ 74 at < 34 weeks' gestation is associated with a 12‐fold increase in the odds of placental abruption compared with lower cut‐offs. Furthermore, pregnant women with an elevated sFlt‐1/PlGF ratio are more likely to experience severe placental insufficiency, which contributes to higher rates of fetal growth restriction^29^. When the sFlt‐1/PlGF ratio exceeded the cut‐off used in our study, especially before 34 weeks, we observed a significantly higher risk of SGA fetuses, leading to increased rates of admission to the NICU.

Therefore, we propose GA‐specific sFlt‐1/PlGF thresholds for predicting PE development and adverse maternal–fetal outcomes. Exceeding the threshold should trigger intensified monitoring and preparedness for preterm delivery, including fetal lung maturation or antenatal transfer^30^, ^31^, ^32^, owing to the high risk of placental dysfunction and fetal compromise.

### Limitations

This single‐center study validated the predictive thresholds of sFlt‐1/PlGF ratio in an independent cohort, which may limit generalizability. The use of lower sFlt‐1/PlGF ratio cut‐offs in our study compared with those used in previous studies probably reflects cohort‐specific variations in study design, population characteristics and PE heterogeneity. Future validation in multicenter studies with larger sample sizes is necessary to improve the generalizability and robustness of our findings across diverse populations.

### Conclusions

This large, prospective cohort study established validated, GA‐specific diagnostic sFlt‐1/PlGF ratio cut‐offs for predicting early‐onset PE and late‐onset PE within 1 week after sFlt‐1 and PlGF measurement in women with high blood pressure from Southern China. Notably, the optimized sFlt‐1/PlGF ratio of ≥ 74 when measured at < 34 weeks demonstrated excellent diagnostic performance for early‐onset PE. The validated thresholds of 74 measured at GA < 34 weeks and 95 measured at GA ≥ 34 weeks not only enabled earlier identification of PE but also showed significant associations with adverse maternal and perinatal outcomes. By specifically targeting pregnant women with high blood pressure, the findings of this study are well aligned with clinical decision‐making needs and have direct clinical implications.

## Supporting information


**Table S1** Soluble fms‐like tyrosine kinase‐1/placental growth factor ratio threshold and sensitivity at fixed specificities for early‐onset pre‐eclampsia diagnosis in derivation cohort.

**Table S2** Soluble fms‐like tyrosine kinase‐1/placental growth factor ratio threshold and sensitivity at fixed specificities for late‐onset pre‐eclampsia diagnosis in derivation cohort.

## Data Availability

The data that support the findings of this study are available on request from the corresponding author. The data are not publicly available due to privacy or ethical restrictions.
