# Supplementary material for: sFlt‐1/PlGF ratio thresholds for diagnosing pre‐eclampsia in pregnant women with high blood pressure
Source: Ultrasound Obstet Gynecol. 2025 Sep 24;66(5):631–40. doi: 10.1002/uog.70075 (PMC12579772; doi:10.1002/uog.70075)
Supplement: Supplementary file 1 — Table S1 Soluble fms‐like tyrosine kinase‐1/placental growth factor ratio threshold and sensitivity at fixed specificities for early‐onset pre‐eclampsia diagnosis in derivation cohort. [file UOG-66-631-s002.docx]

**Table S1** Soluble fms-like tyrosine kinase-1/placental growth factor ratio threshold and sensitivity at fixed specificities for early-onset pre-eclampsia diagnosis in derivation cohort.

| *Fixed Specificity (%)* | *Sensitivity (%) (95% CI)* | *Cut-off value* |
| --- | --- | --- |
| 99 (FPR=1%) | 55.9(46.1 - 65.3) | 283 |
| 97 (FPR=3%) | 66.7(57.1 - 75.3) | 196 |
| 95 (FPR=5%) | 86.5(59.5- 99.1) | 98 |
| 90 (FPR=10%) | 98.2(79.8 - 100.0) | 42 |
| 85 (FPR=15%) | 99.1(95.1 - 100.0) | 30 |

LOPE: Early Onset Pre-Eclampsia; FPR: False Positive Rate; CI: confidence intervals.
