# Supplementary material for: sFlt‐1/PlGF ratio thresholds for diagnosing pre‐eclampsia in pregnant women with high blood pressure
Source: Ultrasound Obstet Gynecol. 2025 Sep 24;66(5):631–40. doi: 10.1002/uog.70075 (PMC12579772; doi:10.1002/uog.70075)
Supplement: Supplementary file 2 — Table S2 Soluble fms‐like tyrosine kinase‐1/placental growth factor ratio threshold and sensitivity at fixed specificities for late‐onset pre‐eclampsia diagnosis in derivation cohort. [file UOG-66-631-s001.docx]

**Table S2** Soluble fms-like tyrosine kinase-1/placental growth factor ratio threshold and sensitivity at fixed specificities for late-onset pre-eclampsia diagnosis in derivation cohort.

| *Fixed Specificity (%)* | *Sensitivity (%) (95% CI)* | *Cut-off value* |
| --- | --- | --- |
| 99 (FPR=1%) | 22.5(16.6 - 29.3) | 216 |
| 97 (FPR=3%) | 45.5(38.0 - 53.1) | 123 |
| 95 (FPR=5%) | 60.1(52.5 - 67.4) | 95 |
| 90 (FPR=10%) | 72.5(65.3 - 78.9) | 69 |
| 85 (FPR=15%) | 79.8 (73.1 - 85.4) | 53 |

LOPE: Late Onset Pre-Eclampsia; FPR: False Positive Rate; CI: confidence intervals.
